# Analysis of Sports Injury Prevalence and Patterns in Recreational Sports Activities in South Korea: Applying the Association Rule Method

**DOI:** 10.3390/life15050701

**Published:** 2025-04-26

**Authors:** Byeong Seok Min, Nara Jang

**Affiliations:** 1 Sociology of Sport, Sunmoon University, Asan 31460, Chungcheongnam-do, Republic of Korea; mbs1208@hanmail.net; 2 Department of Physical Education, Korea National Sport University, Seoul 05541, Republic of Korea

**Keywords:** sports injury, association rule method, recreational sports

## Abstract

This study aims to identify the prevalence and patterns of sports injuries in recreational sports activities in South Korea. This study utilized data from the “survey of safety accidents” conducted by the Korea Sports Safety Foundation and finally, 3182 recreational sports participants who experienced injuries were selected for the study. For data processing, data related to recreational sports injuries were first collected and organized using Excel 2015, and frequency analysis was conducted using the SPSS 25.0 program. Furthermore, the association rule method was applied via Python 3.13.3 to analyze the patterns of injury sites and types. First, by investigating the prevalence of injuries in recreational sports, it was found that the injury frequency was highest in soccer, followed by cycling, hiking, and badminton. Second, in soccer, it was found that when ankle injuries, which have a high injury frequency, occur, knee, toe, and sprain injuries also occur together (Lift: 1.843). Additionally, in cycling, when knee injuries occur, toe, sprain, and strain (bruise) injuries also occur together (Lift: 2.420). In mountain biking, when ankle injuries, which have a high injury frequency, occur, cuts, sprains, stab wounds (cuts), sprains, and strains (bruises) also occur together (Lift: 1.808). The current survey on recreational sports injuries is expected to be used as basic data to prevent injuries in advance for participants in recreational sports, and it is expected that this will allow them to participate in sports by recognizing common injury sites before participating in sports.

## 1. Introduction

In modern society, recreational sports are increasingly recognized as a significant factor in improving the quality of life [1]. As urbanization and technological advancements make lives busier and more stressful around the world, recreational sports are becoming an important way to promote not only physical health but also mental well-being [2]. Recreational sports are more than just a hobby; they play an essential role in helping individuals form and maintain social relationships, boost self-confidence, and effectively manage stress. Therefore, recreational sports have been emphasized as an essential component of leading a healthy and happy life, as well as balancing the overall life of a modern person.

In South Korea, the amount of time modern people spend on leisure is changing, and in fact, the national statistics site (kosis.kr/index) of Statistics Korea reports an increase in the amount of time spent on leisure in 2023 compared to the past [3]. With much leisure time, people are engaged in a variety of activities, and participation in sports is one of the most important contributors to their quality of life and self-esteem. As a result, many scholars and researchers have emphasized the importance of participation in recreational sports, and efforts are being made to increase participation in recreational sports [4].

In recreational sports, ensuring sports safety is of paramount importance, as it is crucial for continued participation. Sports safety refers to minimizing the risk of injury to participants, and a sports injury is defined as “a condition in which an athlete who sustains an injury during competition or training is medically evaluated by a doctor or trainer and is deemed unable to participate in sports for more than one day” [5]. Therefore, even in recreational sports, efforts to prevent injuries are essential.

To prevent sports injuries, it is crucial to identify potential injuries in advance. Since each sport is associated with a distinct set of injuries that affect different parts of the body, identifying the most common injuries beforehand can aid in the planning of preventive measures. Furthermore, as noted by [6], the causes of sports injuries are often difficult to pinpoint due to the variety of situations in which they occur; however, identifying risk factors is necessary for preventing injuries proactively. As such, research on injuries in recreational sports is needed.

Most previous studies have focused on injuries in professional or elite sports [7,8]. However, with the growth of recreational sports, more individuals are becoming involved and expressing interest in these activities, which in turn increases the significance of safety measures in recreational sports. Additionally, due to the interconnection between the development of recreational sports and the advancement of professional elite sports, as well as the enhancement of public quality of life and health through recreational sports, research on injuries in recreational sports has become a critical area of study [9]. Consequently, many scholars have emphasized the necessity of developing strategies and programs to prevent sports injuries and ensure safety.

In efforts to improve sports safety and reduce injuries, various studies have been conducted on the development of sports safety manuals, surveys on sports safety, and sports injuries by sport. However, most of these studies provide only basic information through frequency analysis. In reality, it remains challenging to identify patterns regarding which injuries tend to occur together in specific sports. Given the nature of sports, certain areas of the body are more prone to injury than others. Therefore, it is essential to correlate the injured body parts in the analysis of sports injuries.

To identify patterns in sports injuries, the association rule methodology can be employed. The association rule method is an unsupervised learning technique that uncovers relationships between items [10] and is often referred to as market basket analysis in the domain of consumer behavior. This method serves as a powerful tool for identifying and analyzing relationships between variables within datasets, and it is widely utilized across various fields. The results are typically presented in the intuitive form of “if A occurs, then B also occurs,” facilitating the discovery of latent patterns and associations within the data. Furthermore, the method is capable of extracting frequently occurring rules from large datasets, which aids in the identification of significant trends or patterns. Particularly, it is well suited for analyzing diverse data types, as it accommodates both numerical and categorical data, offering valuable insights in sectors such as marketing, healthcare, and sports. As a result, the association rule methodology has become a critical analytical technique for deriving meaningful insights in research, including the analysis of injury patterns. The method has been applied in sports to uncover patterns, with studies investigating aspects such as scoring patterns, points deducted, and psychological anxiety in athletes [11,12]. Therefore, it holds significant potential for identifying injury patterns among recreational sports participants.

Therefore, this study aims to identify the prevalence and patterns of sports injuries in recreational sports activities in South Korea. Specifically, the frequency of injury by sport was calculated by participation in recreational sports activities in South Korea. Furthermore, the injury patterns of the top three sports with the most frequent injuries were identified in terms of injury site and type. This can be used as basic information for performing pre-stretching or taping to prevent injuries by checking injury information for recreational sports and identifying common injury areas by sport.

## 2. Research Method

### 2.1. Data Collection and Subjects

This study aimed to investigate the prevalence and patterns of injuries associated with participation in recreational sports. For this purpose, secondary data from the Survey of Safety Accidents conducted by the Korea Sports Safety Foundation, a nationally accredited institution, were utilized. Specifically, the study employed data from the Awareness Survey, one of the two components of the overall survey (the other being the In-depth Survey). The Awareness Survey targeted South Korean adults aged 19 years or older who were capable of expressing their subjective opinions. Participants were selected through a stratified sampling method based on demographic variables such as region, gender, and age, ensuring a representative distribution of the national population. The final analytical sample consisted of 3,182 individuals who had experienced injuries while participating in recreational sports. Responses deemed insincere—such as missing values or extreme outliers—were excluded during the data cleaning process. Of the respondents, 1614 were male (50.7%) and 1568 were female (49.3%). The distribution by age group was as follows: 20 s (*n* = 616, 19.4%), 30 s (*n* = 656, 20.6%), 40 s (*n* = 795, 25.0%), 50 s (*n* = 785, 24.7%), and 60 years or older (n = 246, 10.3%), with individuals in their 40 s comprising the largest proportion (Table 1).

It is important to note that this study did not involve the primary collection of data by the authors. Instead, it utilized publicly available secondary data provided by the Korea Sports Safety Foundation. As such, the figure of 3182 respondents represents a refined dataset selected according to the foundation’s internal criteria. Details regarding the original number of individuals contacted or the overall response rate were not disclosed in the public dataset. The data used in this study were obtained from the Korea Sports Safety Foundation’s official website (https://www.sportsafety.or.kr/front/main.do (accessed on 20 December 2023)).

### 2.2. Selection and Content of Survey Variables

In this study, variables related to sport-specific injuries were selected from the Survey of Safety Accidents conducted by the Korea Sports Safety Foundation. Specifically, the survey items used were “Please describe all injuries you experienced in the past year. First, where was the injury site?” and “What type of injury did you sustain at the reported site?” Based on the responses, injury sites were classified into 38 distinct categories, and injury types were categorized into 13 different types. The detailed results are presented in Table 2.

### 2.3. Data Processing Methods

For data processing, data related to recreational sports injuries were first collected and organized using Excel 2015, and frequency analysis was conducted using the SPSS 25.0 program. Furthermore, the association rule method was applied via Python 3.13.3 to analyze the patterns of injury sites and types. Specifically, the association rule method applied the Apriori algorithm, which organized a set of frequent items based on their minimum support and derived rules from within the frequent items. The association rule calculated the support, confidence, and improvement indices, and it should be noted up front that the minimum support was set low to account for the responded characteristics. In association rule mining, the minimum thresholds for “support” and “confidence” usually depend on the characteristics of the dataset, the research objectives, and the problem being analyzed. These values are often compared relatively within the dataset itself [13]. Only patterns with an improvement of at least 1.00 on items that satisfied the minimum support (0.01) and minimum confidence (0.20) were finally adopted. This was a criterion for discovering association rules among many combinations of rules, where an enhancement value of 1.00 or higher meant that the probability of two items co-occurring was higher than the expected probability. The support, confidence, and improvement are calculated using Equation (1).

(1) $$\mathrm{Support}\;\left(A,B\right)=P\left(A\cap B\right)\;\mathrm{Confidence}\;\left(A\to B\right)=\frac{P\left(A\cap B\right)}{P\left(A\right)}\;\mathrm{Lift}=\frac{P\left(B|A\right)}{P\left(B\right)}=\frac{P\left(A\cap B\right)}{P\left(A\right)P\left(B\right)}=\frac{\mathrm{Confidence}}{P\left(B\right)}$$

## 3. Results

### 3.1. Status of Injuries from Participation in Sports Activities by Leisure Sport

Table 3 shows the prevalence of injuries related to participation in leisure activities among South Koreans. The results showed that soccer (439 injuries, 18.2%) led to the highest number of injuries in sports participation, followed by cycling (305 injuries, 12.6%) and hiking (289 injuries, 12.0%). On the other hand, although not shown in the table below, the recreational sports with the lowest number of injuries were paragliding (3, 0.1%) and kendo (3, 0.1%).

### 3.2. Injury Site Patterns by Participation in Recreational Sports

This study applied the association rule method to identify the patterns of injury sites by participation in recreational sports. The minimum support was set to 0.001 and the minimum confidence to 0.100. Furthermore, injury site patterns for the three most common sports, soccer, cycling, and mountain biking, were identified.

#### 3.2.1. Patterns of Injury Sites by Participation in Soccer as a Leisure Sport

Table 4 shows the injuries caused by participation in soccer as a leisure sport in South Korea. The results showed that the most common injury site was the ankle (25.0%), followed by the knee (11.0%) and shin (6.7%). To analyze the patterns of injury site and type by participation in soccer, the association rule was applied. Since the main objective of this study was to identify the injury patterns by site and type, the three most common injury sites (ankle, knee, and shin) in soccer were selected and analyzed. The results for the patterns by injury site only showed the top three with the highest improvement. The results showed that ankle injuries had the highest improvement in injury patterns for knees, toes, and sprains. In the knee, the highest injury patterns were for toes, ankles, and sprains, while in the shin, the highest improvement was for toes, sprains, and calves (Table 5).

#### 3.2.2. Patterns of Injury Sites by Participation in Cycling as a Leisure Sport

Table 6 shows the injuries caused by participation in cycling as a leisure sport in South Korea. The results showed that the most common injury site was the knee (8.8%), followed by the ankle (8.5%) and palm (5.5%). To analyze the patterns of injury site and type by participation in cycling, the association rule was applied. The three most common injury sites (knee, ankle, and palm) in cycling were selected and analyzed. The results for the patterns by injury site only showed the top three with the highest improvement. The results showed that knee injuries had the highest improvement in injury patterns for the following injury types: toe, sprain, strain (bruise) and toe, abrasion (scratch), and sprain. Ankles had the highest pattern improvement for abrasion (scratch), sprain, finger and toenail, sprain, strain (bruise), knee, sprain, and finger. Palms had the highest injury pattern improvement for elbow, abrasion (scratch), calf and wrist, abrasion (scratch), and sprain (Table 7).

#### 3.2.3. Patterns of Injury Sites by Participation in Hiking as a Leisure Sport

Table 8 shows the injuries caused by participation in hiking as a leisure sport in South Korea. The results showed that the most common injury site was the ankle (15.2%), followed by the knee (8.1%) and palm (3.7%). To analyze the patterns of injury site and type by participation in hiking, the association rule was applied. The three most common injury sites (ankle, knee, and palm) in hiking were selected and analyzed. The results for the patterns by injury site only showed the top three with the highest improvement. The results showed that ankle injuries had the highest improvement in injury patterns for the following injury types: stab (cut), sprain, strain (bruise), toenail, sprain, strain (bruise), abrasion (scratch), sprain, and stab (cut). Knee had the highest pattern improvement for hip joint, ankle, sprain, thigh (femur), sprain, sole, thigh (femur), ankle, and sole, while palm had the highest injury pattern improvement for sprain, hips, and strain (bruise) (Table 9).

## 4. Discussion

Recreational sports participants are increasingly concerned about sports safety [14]. In particular, the importance of preventing sports injuries has been emphasized, as such injuries can limit the ability to continue engaging in leisure activities. Therefore, in addition to identifying the prevalence of injuries by recreational sports activity, this study also examined patterns in the injury site and type for the top three most common sports. To address this issue, the association rule method was applied, and the resulting discussion and recommendations are presented below.

First, by investigating the prevalence of injuries in recreational sports, the injury frequency was highest in soccer, followed by cycling, hiking, and badminton. Soccer and cycling are among the sports with the highest rates of injuries, both in recreational and elite sports. In fact, a study examining injuries among athletes who participated in the 2012 London Olympics found that taekwondo, soccer, and BMX had the highest injury rates [15]. This suggests that the risk of injury in both recreational and elite levels of soccer and cycling is considerable. The age of participants in hiking was notably higher than in the other sports. Specifically, an analysis of the ages of participants in soccer, cycling, and hiking, based on data from the Korea Sports Safety Foundation, revealed that those in their 40s or older accounted for 39% of participants in soccer, 54% in cycling, and 63% in hiking, indicating that hiking participants were the oldest. Therefore, the older age of participants is likely a contributing factor to the higher frequency of injuries observed in hiking.

Second, patterns of injury site and type for the top three recreational sports with the highest injury frequency were analyzed. In soccer, the most common injury sites were the ankle, knee, and shin. Soccer had the highest number of ankle injuries, as most rapid body movements, landings after jumps, and shooting actions involve the lower extremities and ankles [16,17]. Furthermore, ankle injuries were most commonly sprains, with knee injuries frequently co-occurring. This finding is supported by [18], which reported that ankle sprains are common among soccer players due to the prolonged physical activity involved. Ref. [19] also noted that ankle and knee injuries are prevalent in the lower extremities. Additionally, ref. [20] further emphasizes that ankle injuries are the most common in soccer, underscoring the need for continued research on ankle and lower extremity injuries in this sport.

In cycling, injuries were most common in the knees, ankles, and palms. Cycling, a sport that involves pedaling a bicycle, places significant stress on the knees. However, the most relevant injury sites and types associated with knee injuries were toes, sprains, and strains. While sprains may not be particularly common, reports indicate that knee injuries can alter lower extremity alignment and stability, potentially decreasing ankle stability, which could explain this finding [21]. Moreover, patterns in injury sites and types in cycling can also be identified by examining ankle and palm injuries [22].

Finally, in hiking, the most common injury sites were ankle, knee, and palm. Hiking is one of those recreational sports that is more about improving health through climbing than competing. Nevertheless, cuts from tree branches and sprains and strains from uneven trails were the most common patterns in hiking. In hiking, most injuries were not caused by opponents but by the external environment and their own movement. In the future, it would be more meaningful to look at injuries in hiking by age. When looking at the characteristics of the sports with the highest rates of injury in recreational sports participation in general, the majority were lower extremity sports. They were also considered sports that were practiced over a long period, although the amount of time spent in the sport varied. Therefore, there is a need for some time restrictions in recreational sports activities and a need to identify the injuries that occur in the lower extremities and find ways to prevent them. 

Finally, this study identified the prevalence and patterns of injuries associated with participation in recreational sports activities. However, this study was limited by the fact that it only looked at the site and type of injury to identify injury patterns without accounting for various other variables. It also failed to reflect the demographic characteristics of the participants. However, this study has several limitations. First, in analyzing injury patterns, it only considered the site and type of injury, without including various factors such as training intensity, competition level, player position, and environmental conditions. Second, the study did not reflect the demographic characteristics of the participants, failing to consider the influence of age, gender, and athletic experience on injury occurrence. Third, since the analysis was based on general injury data rather than focusing on specific sports, it was limited in clearly identifying injury patterns across all sports disciplines. To address these limitations, future research should incorporate a wider range of variables and conduct more in-depth analyses that consider the characteristics of specific sports. It is still significant in terms of identifying injuries to recreational sports activities. Furthermore, the patterns in the site and type of injuries could be used as a basis for developing programs on warm-up exercises and treatment to proactively prevent injuries.

## 5. Conclusions

The findings of this study highlight the prevalence and patterns of injuries in recreational sports. Soccer exhibited the highest injury frequency, followed by cycling, hiking, and badminton. The most commonly affected body parts were the ankle in soccer, the ankle, knee, and palm in hiking, and the knee, ankle, and palm in cycling. These results provide essential insights into injury patterns, serving as valuable data for developing injury prevention strategies and enhancing safety measures in recreational sports participation.

## Figures and Tables

**Table 1:** Demographic characteristics of the study participants.

Category		N	%
Gender	Male	1614	50.7
	Female	1568	49.3
Age	20s	616	19.4
	30s	656	20.6
	40s	795	25.0
	50s	785	24.7
	60s and older	246	10.3

**Table 2:** Injury site and responses from the injury survey.

Item 1. Please Tell Me About Any Injuries You Have Had in the Past Year. First, Where Was the Injury Site?									
No.	Response	No.	Response	No.	Response	No.	Response	No.	Response
1	Scalp	9	Cheekbone/cheek	17	Upper arm	25	Hip joint	33	Ankle
2	Eye	10	Head	18	Elbow	26	Hip	34	Sole
3	Nose	11	Neck (cervical)	19	Lower arm	27	Thigh (femur)	35	Instep
4	Mouth	12	Chest	20	Wrist	28	Groin	36	Toe
5	Teeth	13	Stomach	21	Palm	29	Knee	37	Toenail
6	Ear	14	Back	22	Back of hand	30	Shin	38	Other
7	Forehead	15	Lower back	23	Finger	31	Calf		
8	Chin	16	Shoulder	24	Nail	32	Achilles tendon		
Item 2. What Type of Injury Did You Have in the Injury Site?									
No.	Response	No.	Response	No.	Response	No.	Response	No.	Response
1	Sprain	4	Laceration (tear)	7	Bleeding	10	Pain	13	Other
2	Fracture	5	Cut	8	Strain (bruise)	11	Inflammation (festering)		
3	Dislocation (falling off)	6	Abrasion (scratch)	9	Stab (cut)	12	Rupture (burst)		

**Table 3:** Injury status by participation in recreational sports in South Korea (top 10 sports).

Rank	Sport	Injury Frequency	Injury Rate	Rank	Sport	Injury Frequency	Injury Rate
1	Soccer	439	18.2	6	Bowling	125	5.2
2	Cycling	305	12.6	7	Bodybuilding	124	5.1
3	Hiking	289	12.0	8	Skiing/snowboarding	103	4.3
4	Badminton	175	7.2	9	Golf	96	4.0
5	Basketball	125	5.2	10	Foot volleyball	81	3.4

**Table 4:** Analysis of injury site frequency by participation in soccer (top 5).

Rank	Site	Frequency	%
1	Ankle	250	25.0
2	Knee	110	11.0
3	Shin	67	6.7
4	Calf	50	5.0
4	Toe	50	5.0

**Table 5:** Analysis of injury site and type patterns by participation in soccer (top 3 in improvement).

Antecedents	Consequents	Support	Confidence	Lift
Top 3 ankle injuries_improvement				
Knee, toe, sprain	Ankle	0.021	0.909	1.843
Knee, toe, strain (bruise)	Ankle	0.017	0.888	1.802
Knee, abrasion (scratch), toe	Ankle	0.015	0.875	1.774
Top 3 knee injuries_improvement				
Toe, ankle, sprain	Knee	0.039	0.500	2.304
Abrasion (scratch), ankle, toe	Knee	0.029	0.466	2.150
Toe, sprain, strain (bruise)	Knee	0.029	0.466	2.150
Top 3 shin injuries_improvement				
Toe, sprain, calf	Shin	0.019	0.600	4.540
Abrasion (scratch), ankle, strain (bruise)	Shin	0.067	0.294	2.225
Abrasion (scratch), sprain, strain (bruise)	Shin	0.067	0.294	2.225

**Table 6:** Analysis of injury site frequency by participation in cycling (top 5).

Rank	Site	Frequency	%
1	Knee	105	8.8
2	Ankle	102	8.5
3	Palm	66	5.5
4	Shin	43	3.6
5	Elbow	37	3.1

**Table 7:** Analysis of injury site and type patterns by participation in cycling (top 3 in improvement).

Antecedents	Consequents	Support	Confidence	Lift
Top 3 knee injuries_improvement				
Toe, sprain, strain (bruise)	Knee	0.019	0.833	2.420
Toe, abrasion (scratch), sprain	Knee	0.019	0.833	2.420
Toe, ankle, abrasion (scratch)	Knee	0.016	0.800	2.323
Top 3 ankle injuries_improvement				
Abrasion (scratch), sprain, finger	Ankle	0.013	1.000	2.990
Toenail, sprain, strain (bruise)	Ankle	0.019	1.000	2.990
Ankle, sprain, finger	Ankle	0.013	1.000	2.990
Top 3 palm injuries_improvement				
Elbow, abrasion (scratch), calf	Palm	0.019	0.666	3.080
Wrist, abrasion (scratch), sprain	Palm	0.019	0.666	3.080
Elbow, abrasion (scratch), sprain	Palm	0.032	0.600	2.772

**Table 8:** Analysis of injury site frequency by participation in hiking (top 5).

Rank	Site	Frequency	%
1	Ankle	157	15.2
2	Knee	84	8.1
3	Palm	38	3.7
4	Calf	35	3.4
5	Sole	25	2.4

**Table 9:** Analysis of injury site and type patterns by participation in hiking (top 3 in improvement).

Antecedents	Consequents	Support	Confidence	Lift
Top 3 ankle injuries_improvement				
Stab (cut), sprain, strain (bruise)	Ankle	0.010	1.00	1.808
Toenail, sprain, strain (bruise)	Ankle	0.017	1.00	1.808
Abrasion (scratch), sprain, stab (cut)	Ankle	0.017	1.00	1.808
Top 3 knee injuries_improvement				
Hip joint, ankle, sprain	Knee	0.010	1.00	3.380
Thigh (femur), sprain, sole	Knee	0.010	1.00	3.380
Thigh (femur), ankle, sole	Knee	0.010	1.00	3.380
Top 3 palm injuries_improvement				
Sprain, hips, strain (bruise)	Palm	0.017	0.600	4.484
Abrasion (scratch), hips, strain (bruise)	Palm	0.021	0.500	3.736
Laceration (tear), ankle, sprain	Palm	0.021	0.500	3.736

## Data Availability

The datasets used and/or analyzed during the current study are available from the corresponding author upon reasonable request.
